# A Complex Microbial Interplay Underlies Recurrent Vulvovaginal Candidiasis Pathobiology

**DOI:** 10.1128/mSystems.01066-21

**Published:** 2021-10-19

**Authors:** Nicolas Papon, Patrick Van Dijck

**Affiliations:** a Univ Angers, Univ Brest, GEIHP, SFR ICAT, Angers, France; b Laboratory of Molecular Cell Biology, Institute of Botany and Microbiology, KU Leuven, Leuven-Heverlee, Belgium; c VIB-KU Leuven Center for Microbiology, Leuven, Belgium

**Keywords:** vulvovaginitis, *Candida*, *Lactobacillus*, microbial interaction, biofilm, probiotics

## Abstract

While extremely prevalent, painful, and difficult to treat, vulvovaginal candidiasis remains largely understudied in the field of women’s health. In a recent issue of *mSystems*, McKloud et al. (E. McKloud, C. Delaney, L. Sherry, R. Kean, et al., mSystems 6:e00622-21, 2021, https://doi.org/10.1128/mSystems.00622-21) shed light on a pivotal role of a complex Candida-Lactobacillus interplay that may regulate the pathophysiology of recurrent vulvovaginal candidiasis (RVVC). This advancement not only gives new insight into the molecular mechanisms governing interkingdom interactions modulating RVVC disease, but also provides evidence that probiotic *Lactobacillus*-based therapeutic approaches could be efficient for fighting these problematical fungal infections.

## COMMENTARY

**FIG 1:**
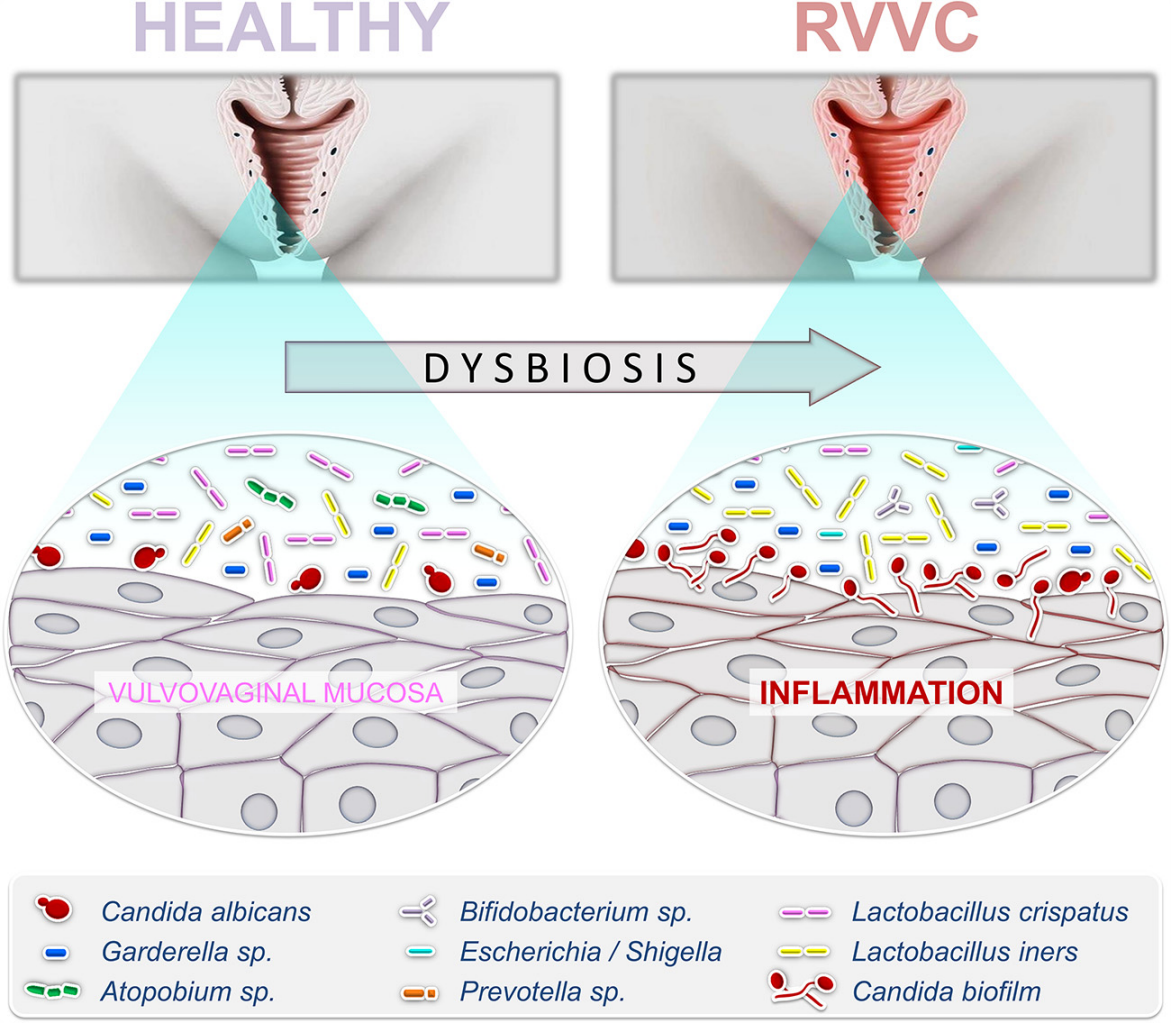
Complex microbial interplay underlies recurrent vulvovaginal candidiasis pathobiology. A complex microbiome is associated with the healthy vulvovaginal mucosa. Broad-spectrum antibiotic treatment, high-estrogen-containing contraceptives, sexual activity, pregnancy, and uncontrolled diabetes mellitus can induce a global disruption of the equilibrium in these microbial communities (termed dysbiosis) and promote the overgrowth of *Candida* yeast, leading to vulvovaginitis candidiasis (VVC) onset and subsequent recurrent VVC (RVVC). Pathobiology consists of a strong inflammation of the mucosa at the origin of typical symptoms such as burning, itching, white clumpy discharge, and redness of the genital track. McKloud and colleagues (5) provide unprecedented insight into the complex microbial interplay underlying RVVC. Notably, they show that the bacterium Lactobacillus crispatus is able to condition a healthy vaginal environment by reducing Candida albicans load. In contrast, observation of RVVC samples revealed increased levels of Lactobacillus iners—which has been shown previously to be indicative of vaginal dysbiosis—together with an extinction of specific *Lactobacillus* species that are reported to be usually associated with a healthy vaginal microbiome, notably L. crispatus. This may indicate that potential protection from *Candida* local overgrowth can be provided by resident lactobacilli in the vaginal microenvironment.

If you asked the general public to provide a single word regarding what they know about “infectious microorganisms,” the majority of individuals would offer the terms “viruses” or “bacteria,” and sometimes “parasites,” but rarely “fungi.” Factually, the public awareness of fungi as potentially dangerous pathogens is low. Yet, fungi are a major problem in the clinical setting, with an estimated 1.5 million deaths resulting from invasive fungal infections each year, a mortality rate three times greater than those for breast cancer, influenza, or malaria (1). Furthermore, fungi infect billions of people every year, and roughly a quarter of the world population suffers from superficial fungal infections. This is particularly the case for vulvovaginal candidiasis (VVC), which represents the second most common cause of vaginitis after bacterial vaginosis (2). The prevalence of this disease affecting the lower female reproductive tract is puzzling; VVC is diagnosed at least one time in the life of up to two-thirds of healthy women. Beyond these epidemiological considerations, it must be noted that VVC is strongly uncomfortable for women due to typical symptoms such as burning, itching, white clumpy discharge, and redness of the vulva and vagina, not to mention the resulting dyspareunia. It is now well documented that broad-spectrum antibiotic treatment, high-estrogen-containing contraceptives, sexual activity, pregnancy, and uncontrolled diabetes mellitus are among the prominent risk factors for VVC (2). From a pathophysiological point of view, it is thought these factors likely result in alterations in the vulvovaginal microenvironment that in turn promotes *Candida* yeast overgrowth and symptomatic infection. In the case of a single episode of VVC, the disease can be often resolved thanks to a local treatment (vaginal ovules), usually based on imidazole antifungals, or through a single oral treatment with fluconazole. However, the therapeutic approach is much more delicate for women suffering from recurrent VVC (RVVC), which is defined as developing the disease up to four times a year. This recurrent form, which affects more than hundred million women worldwide, remains a real health calamity to manage for practitioners (3). The treatment is in this case systemic, using triazoles (mainly fluconazole, or alternatively, itraconazoles) coupled with a local application of imidazoles. Unfortunately, therapeutic failures are usually observed, and, in other cases, the treatment tends to only transiently reduce the symptoms during the treatment course. As a consequence, it is essential to pursue sustained research to decipher the pathophysiology of RVVC. More specifically, there is a particular interest in characterizing the complex interactions that may occur between *Candida* yeasts, bacterial communities of the microflora, and epithelial and immune cells residing in the vaginal mucosa, because this knowledge could help in developing new strategies for early diagnoses and better targeting of antifungal therapeutic interventions (4). As such, the study led by the Gordon Ramage research group and recently published in *mSystems* provides an unprecedented insight into the fluctuations in antagonistic interkingdom interactions between *Candida* and *Lactobacillus* associated with RVVC disease (5) (Fig. 1).

In this report, McKloud and colleagues took advantage of a panel of 100 clinical isolates (40 recovered from RVVC and 60 from healthy women) to investigate fungal influence and changes in bacterial communities and also to analyze potential microbial interplays contributing to RVVC pathobiology (Fig. 1). The first series of experiments suggest that *Candida* burden could be a contributing factor to disease pathology and an indicator for VVC onset and subsequent recurrence. Indeed, the *Candida* load was found to be significantly lower in healthy samples than that in RVVC samples. Interestingly, the fungal load appeared to be primarily unaccompanied by a bacterial burden. In addition, consistent with previous studies, the authors show that the microbial communities present during RVVC are similar, at the phylum and genus levels, to those found in healthy women, detecting a *Lactobacillus*-dominated population with prominent vaginal anaerobes such as Gardnerella, Prevotella, and Atopobium (6) (Fig. 1). However, one of the main findings of this study consisted of the observation in RVVC samples of increased levels of Lactobacillus iners—which has been shown previously to be indicative of vaginal dysbiosis—together with an extinction of specific *Lactobacillus* species that are reported to be usually associated with a healthy vaginal microbiome, notably the l-lactic acid and H_2_O_2_ producers Lactobacillus crispatus and Lactobacillus jensenii (6) (Fig. 1). This may indicate, as previously suggested, that potential protection from local overgrowth of *Candida* can be provided by resident lactobacilli in the vaginal microenvironment (7).

Further assessments of the cohort also revealed some key facts that are commonly, but unfortunately, observed in gynecology units and by practitioners (8). In this respect, the results published by McKloud and colleagues confirm first that contraceptive devices are more dysbiotic for the vulvovaginal microbial communities compared to hormonal contraceptives. Above all, the results also point out that even though a recent antifungal regimen transiently restored the microbial profile to a state similar to those of healthy women, the currently available antifungal regimens are not sufficient to definitively restore the microbiome to a health-like state, and this, obviously, may condition the recurrence of VVC in these patients. This is, however, not so intriguing, insofar as it was previous demonstrated that a transition from disease to an intermediate state only is observed in women with VVC following a classical fluconazole oral treatment (9).

Biofilm formation by *Candida* isolates has long been considered a pathogenicity factor but also a main mechanism for antifungal tolerance (10), regardless of the type of candidiasis (deep seated, skin, or mucosal). However, specifically in the context of RVVC, the presence of biofilms during the disease was hitherto a matter of debate. In this study, Candida albicans was found to account for 73% of the *Candida* species isolated in women with RVVC, while other non-*albicans Candida* (NAC) species, such as Candida glabrata, Candida dubliniensis, Candida parapsilosis, and Candida krusei, accounted for the remaining 27%. With the exception of C. glabrata, which despite its lack of hyphal growth forms clear biofilms, it is now well established in the literature that the other NAC species display little or no biofilm formation capacity *in vivo* and *in vitro* (11, 12). Here, the Gordon Ramage group has (i) systemically observed heterogeneous biofilm formation in C. albicans RVVC isolates, (ii) clearly visualized C. albicans hyphae and aggregates in lavage fluids from women with RVVC, and (iii) detected upregulation of C. albicans biofilm-associated genes in these samples. This was not observed in samples recovered from women with RVVC and NAC isolates. Overall, these data thus suggest that *Candida* biofilm formation on the genital epithelium is a potential cause of treatment failure and likely contributes to subsequent recurrence of VVC. It is also likely that the intrinsic tolerance against azoles observed in several NAC species (such as C. glabrata or C. krusei) prevents them from being cleared by the antifungal treatment (13).

In a last series of experiments, McKloud and colleagues have characterized the apparent antagonism of lactobacilli and C. albicans at a molecular level. For instance, the species Lactobacillus rhamnosus, which is well known for its potential as a probiotic, was shown here to downregulate C. albicans biofilm-related gene expression when cocultured. In contrast, Lactobacillus iners, which was previously demonstrated to be more abundant in the RVVC microbiome, resulted in the upregulation of most of these genes. Finally, a series of exciting new data was obtained by studying the transcriptional reprogramming that underlies the antagonism of Lactobacillus crispatus and C. albicans. Coculture and transcriptomic analysis revealed that L. crispatus triggers amino acid acquisition from C. albicans and may enable suppression of lactate-associated virulence factors in the yeast.

In conclusion, this enlightening new report by the Gordon Ramage group provides unprecedented insight into a complex microbial interplay underlying RVVC (Fig. 1). Notably, they confirm that the RVVC pathophysiology mostly relies on C. albicans-specific attributes (hyphal morphogenesis, biofilm formation, and pathogenesis) that differentiate it from other prominent NAC species, as recently revealed within the framework of a study deciphering the distinct pathogenicity patterns of *Candida*, which are defined by highly species-specific transcriptional profiles during infection of vaginal epithelial cells (14). Above all, it must be highlighted that the present clinical study provides an elegant demonstration of the capacity of L. crispatus to condition a healthy vaginal environment by modulating gene expression in C. albicans and thereby reducing fungal load in this highly complex biofilm model. Interestingly, it was recently revealed in a model of oropharyngeal candidiasis that Lactobacillus johnsonii may dampen C. albicans virulence both by inhibiting yeast development and by preventing burden of potentially synergistic enterococci (15). In addition, the study confirms the complex interplay between microbial species resulting in growth and virulence modulation, as demonstrated, for example, for lactobacilli, Enterococcus faecalis, and C. albicans in the gastrointestinal tract (16). Taken together, all of these recent advancements pinpoint the pivotal role of specific *Lactobacillus* species in modulating *Candida* proliferation on various human mucosae. Beyond this essential and new knowledge concerning the pathophysiology of mucosal candidiasis, these advancements definitively propel promising avenues for improving women’s health through probiotic *Lactobacillus*-based regimens (17).
